# The Fosfomycin Resistance Gene *fosB3* Is Located on a Transferable, Extrachromosomal Circular Intermediate in Clinical *Enterococcus faecium* Isolates

**DOI:** 10.1371/journal.pone.0078106

**Published:** 2013-10-29

**Authors:** Xiaogang Xu, Chunhui Chen, Dongfang Lin, Qinglan Guo, Fupin Hu, Demei Zhu, Guanghui Li, Minggui Wang

**Affiliations:** 1 Institute of Antibiotics, Huashan Hospital, Fudan University, Shanghai, China; 2 Key Laboratory of Clinical Pharmacology of Antibiotics, Ministry of Health, China; University Medical Center Utrecht, The Netherlands

## Abstract

Some VanM-type vancomycin-resistant *Enterococcus faecium* isolates from China are also resistant to fosfomycin. To investigate the mechanism of fosfomycin resistance in these clinical isolates, antimicrobial susceptibility testing, filter-mating, Illumina/Solexa sequencing, inverse PCR and fosfomycin resistance gene cloning were performed. Three *E. faecium* clinical isolates were highly resistant to fosfomycin and vancomycin with minimal inhibitory concentrations (MICs) >1024 µg/ml and >256 µg/ml, respectively. The fosfomycin and vancomycin resistance of these strains could be co-transferred by conjugation. They carried a fosfomycin resistance gene *fosB* encoding a protein differing by one or two amino acids from FosB, which is encoded on staphylococcal plasmids. Accordingly, the gene was designated *fosB3.* The *fosB3* gene was cloned into pMD19-T, and transformed into *E. coli* DH5α. The fosfomycin MIC for transformants with *fosB3* was 750-fold higher than transformants without *fosB3*. The *fosB3* gene could be transferred by an extrachromosomal circular intermediate. The results indicate that the *fosB3* gene is transferable, can mediate high level fosfomycin resistance in both Gram-positive and Gram-negative bacteria, and can be located on a circular intermediate.

## Introduction

Fosfomycin is a small molecule that inhibits the first step in bacterial cell wall synthesis by acting as an analogue of phosphoenolpyruvate 1. It is active against both Gram-positive and Gram-negative bacteria, and has been extensively used therapeutically in many countries 24. In China, fosfomycin alone or in combination with other antibiotics is commonly used in the treatment of infections caused by Gram-positive bacteria, including *Staphylococcus* spp. and *Enterococcus* spp.

Data from the CHINET national bacterial resistance surveillance project showed that the fosfomycin resistance rate in *E. faecium* was 11.3% in China in 2012 (unpublished data). Fosfomycin resistance is caused by plasmid-mediated fosfomycin genes in many bacterial species and also by chromosomal mutations 1, 5, 6. Currently, plasmid-mediated fosfomycin resistance determinants, *fosA*, *fosB* and *fosC*, have been discovered and are related to fosfomycin resistance in *Escherichia coli* (*fosA*, *fosC*) 7, *Enterobacter cloacae* (*fosA*) 8, *Klebsiella pneumoniae* (*fosA*) 9, and *Staphylococcus* spp. (*fosB*) 1, 6. Most chromosomally resistant mutants have an impairment in the L-α-glycerophosphate uptake system (GlpT) or the hexose phosphate uptake system (Uhp) 1, 5. Mutations that diminish the affinity of fosfomycin for its target (MurA) also result in resistance 6. Overexpression of the target protein (MurA) is involved in fosfomycin resistance as well 10.

We previously found VanM-type vancomycin-resistant *E. faecium* strains in China 11. Three of them were also resistant to fosfomycin. To explore the mechanism of fosfomycin resistance in *E. faecium*, we sequenced genomic DNA of the fosfomycin- and vancomycin-resistant clinical *E. faecium* strain HS0661 and discovered the fosfomycin resistance gene, *fosB3*, which was carried by an extrachromosomal circular intermediate. The *fosB3* gene was also detectable in two additional fosfomycin- and vancomycin-resistant clinical *E. faecium* strains and in their transconjugants by PCR.

## Materials and Methods

### Bacterial Strains

Three vancomycin and fosfomycin resistant *E. faecium* clinical strains, HS0661, HS0761 and HS07216, were isolated from patients at a teaching hospital of Fudan University in 2006 and 2007 11. *E. faecium* BM4105RF (Fus^r^, Rif^r^) and *E*. *coli* DH5α (Takara, Dalian, China) were used as recipients in mating experiments and cloning, respectively. *E. faecalis* ATCC29212 was used as a quality control for minimal inhibitory concentration (MIC) determination.

### Antimicrobial Susceptibility Testing

The MICs for seven antimicrobial agents were measured by Etest on Mueller-Hinton agar (Oxoid, Basingstoke, England) with results interpreted according to recommendations of the Clinical and Laboratory Standards Institute (CLSI). Due to the lack of acknowledged fosfomycin breakpoints for *E. faecium*, we used the fosfomycin breakpoints for *E. faecalis* proposed by the CLSI [12].

### Mating Experiment

Filter-mating was carried out with BM4105RF as previously described [13], and transconjugants were selected on brain heart infusion (BHI) agar (Oxoid, Basingstoke, England) plates containing fusidic acid (10 µg/ml) and rifampin (100 µg/ml) for counter-selection, and fosfomycin (1024 µg/ml) and glucose-6-phosphate (25 µg/ml) to select for transferable resistance.

### Solexa Sequencing and Analysis

Genomic DNA was extracted from *E. faecium* HS0661 with the QIAamp DNA mini kit (Qiagen, Germany). Sequencing was performed with an Illumina/Solexa 1G Genome Analyzer. DNA preparation, cluster formation, primer hybridization and DNA amplification reactions were performed according to the manufacturer’s recommended protocol. Sequencing was performed using 75-bp paired-end reads of randomly sheared 200-bp fragments in a single flow cell. Genome scaffold assembly was performed with a Velvet assembler [14]. BLASTN was used to search the NCBI’s nucleotide sequence database for sequences related to that found in strain HS0661. Primers fosBcF (5′AATCGGATTTTAGTGTGGAAACA3′), and fosBcR (5′ GGGTAATCGGATAATAGTGTGGA3′) were used to detect and confirm *fos* gene sequences in clinical strains and their transconjugants. To determine flanking sequences genomic DNA of HS0661 was digested with HindIII, BamHI or SmaI, respectively. Digested DNAs were circularized by self-ligation with T4 DNA ligase (Takara, Dalian, China). The self-ligated DNAs were used as templates for inverse PCR using primers fosBiF (5′TGTCAGCCCCTAAAATATCTCT3′ located within the *fosB3* gene) and fosBiR (5′GTTTCAAATGTACCTAAAGAACT3′ located within the *tnpA* gene).

### Cloning of *fosB3* Gene

To further elucidate the function of *fosB3*, the gene was cloned in *E. coli*. We designed a PCR primer pair, EfosBF (5′CTTTATGGCACCTAAAGTTAGCGA3′) and EfosBR (5′ACCACTAAAATAGGCTCTAATCCT3′), according to sequence flanking fosB3 in HS0661. The target sequence included *fosB3* and 159-bp of upstream sequence that contained two potential promoter-like sequences according to the Neural Network Promoter Prediction program (http://promotor.biosino.org/). The PCR product was cloned into Amp^r^ vector pMD19-T (Takara, Dalian, China). The resulting plasmid, pMD-fosB, was transformed into *E. coli* DH5α. selecting with ampicillin (100 µg/ml), fosfomycin (30 µg/ml) and glucose-6-phosphate (25 µg/ml).

### PFGE and Southern Hybridization Analysis

Pulsed field gel electrophoresis (PFGE) analysis was performed using a CHEF mapper system (Bio-Rad, USA). Agarose plugs were prepared with proteinase K (Merck, Germany) at 1 mg/ml and digested with S1 nuclease or SmaI (Takara, Dalian, China). The digested DNA was subjected to electrophoresis at 6 V/cm, 14°C, in a 1.0% agarose gel (Bio-Rad, USA) with pulse times of 5 to 30 s for 22 h. Banding patterns were interpreted using criteria devised by Tenover et al 15. PFGE after enzyme S1 treatment (S1-PFGE), as described by Barton et al [16], was used to determined plasmid size.

After depurination, denaturation, and neutralization of the gel, DNAs were transferred to a positively charged nylon membrane (Roche, Germany) by a vacuum blotter model 785 (Bio Rad, USA). The membrane was hybridized with *fosB3* or *vanM* probes according to instructions for Dig High Prime DNA Labeling and Detection Starter Kit II (Roche, Germany). Hybridization signals were detected with the ChemiDoc™ XRS+ system (Bio Rad, USA).

### Nucleotide Sequence Accession Numbers

The sequence of the fosfomycin resistance gene *fosB3* from *E. faecium* HS0661 has been assigned GenBank accession number HQ219726. The Illumina/Solexa sequencing dataset has been deposited at the NIH Short Read Archive under accession number PRJNA206216.

## Results

### Antimicrobial Susceptibility and Resistance Transfer by Conjugation

The three *E. faecium* isolates were resistant to fosfomycin, vancomycin, erythromycin, ciprofloxacin, ampicillin and gentamicin, but susceptible to linezolid. The MIC for fosfomycin was >1024 µg/ml and for vancomycin was >256 µg/ml. The vancomycin and fosfomycin resistance of these isolates could be co-transferred by conjugation to *E. faecium* BM4105RF, but the resistance to other drugs could not (Table 1). In thrice repeated mating experiments the conjugation frequencies of HS0661, HS0761 and HS07216 were 1.5±0.8×10^−6^, 1.7±1.0×10^−6^, and 1.6±0.9×10^−7^, respectively.

**Table 1:** Antimicrobial susceptibility and molecular characteristics of *fosB3* positive *E. faecium* clinical strains and BM4105RF transconjugants.

Strains	Antibiotic susceptibility (µg/ml) a							PFGE	*fosB3*
	FOS	VAN	AMP	CIP	ERY	GEN	LZD	Type	gene
Clinical isolate									
HS0661	>1024	>256	>256	>32	>256	>1024	1.5	A	+
HS0761	>1024	>256	>256	>32	>256	>1024	1.5	A	+
HS07216	>1024	>256	>256	>32	>256	>1024	1.5	B	+
Transconjugant									
BM0661-1	>1024	>256	3	1.0	0.125	12	1.0	C	+
BM0661-2	>1024	>256	2	1.0	0.125	12	1.0	C	+
BM0761-1	>1024	>256	3	1.0	0.125	8	1.5	C	+
BM0761-2	>1024	>256	3	1.0	0.125	8	1.5	C	+
BM07216-1	>1024	>256	3	1.0	0.125	12	1.5	C	+
BM07216-2	>1024	>256	3	1.0	0.125	12	1.5	C	+
Recipient									
BM4105RF	48	0.75	3	1.5	0.125	12	1.5	C	NA^b^

a FOS, fosfomycin; AMP, ampicillin; VAN, vancomycin; GEN, gentamicin; ERY, erythromycin; LZD, linezolid; CIP, ciprofloxacin;

b not applicable.

### Analysis of the *fosB3* Gene and its Adjacent Sequence

**Figure 1:**
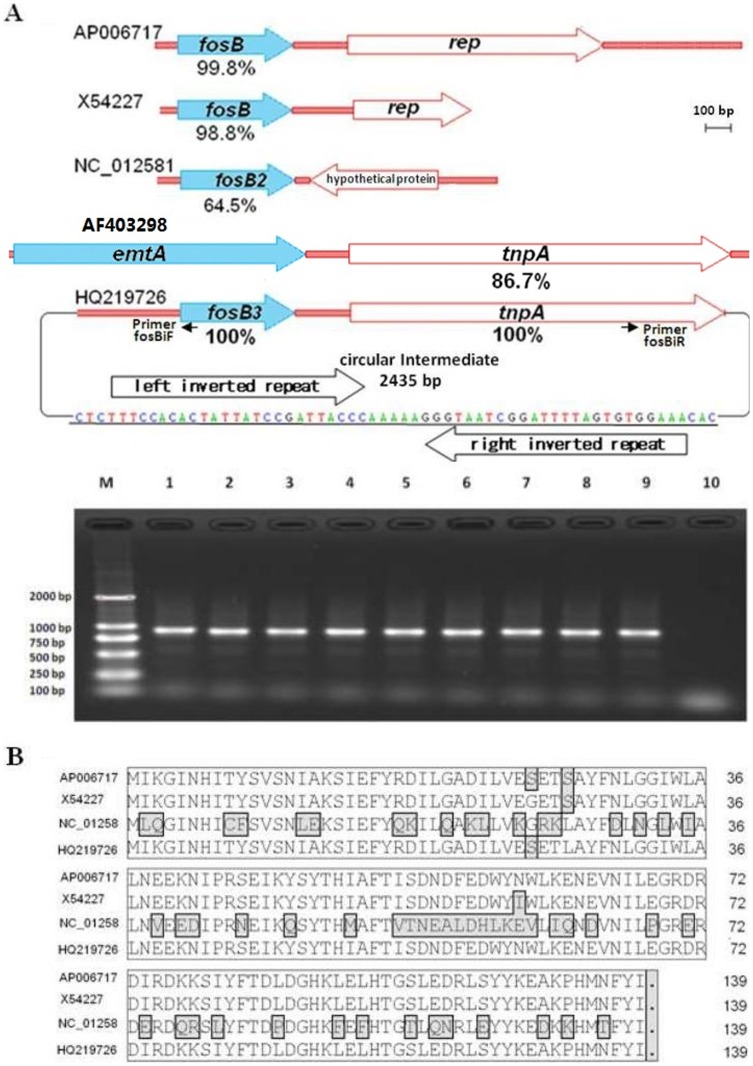
Comparison of the sequences of *fosB* and *tnpA* genes. Alignment of *fosB/tnpA* genes and the gel electrophoresis of inverse PCR products (A). Alignment of the deduced amino acid sequences of *fosB* genes (B). The junction region (underline sequence) found in inverse PCR consists of the left and right inverted repeats. Lanes: M, DL2000; 1–3, *E. faecium* clinical isolates of HS0661, HS0761 and HS07216, respectively; 4 and 5, transconjugants BM0661-1 and BM0661-2; 6 and 7, transconjugants BM0761-1 and BM0761-2; 8 and 9, BM07216-1 and BM07216-2; 10, *E. faecium* BM4105RF. The following sequences were used in this figure: *fosB* (AP006717) in *S. haemolyticus*, *fosB* (X54227) in *S. epidermidis*, *fosB2* (NC_012581) in *Bacillus anthracis*, *tnpA* (AF403298) in *E. faecium* and *fosB3*/*tnpA* (HQ219726) in *E. faecium* HS0661. The residues which differ from the consensus sequence were boxed.

Assembly of the Illumina/Solexa data resulted in 3 Mb of sequence data in 1013 contigs with an N50 size of 9.8 kb. With NCBI BLASTN, a 2,414-bp contig containing a fosfomycin resistance gene was detected. The *fos* gene was 420 bp in length, encoded a 139-amino-acid protein, and had 98.8%–99.8% nucleotide identity with *fosB* genes discovered in plasmids from *Staphylococcus* spp [17], [18]. The enterococcal *fos* gene differed by one amino acid from that found in *S. haemolyticus* and by two amino acids from that found in *S. epidermidis.* The nucleotide sequence and deduced amino acid sequence of the gene had only 64.5% and 60% identity, respectively, with the chromosomally-determined *fosB2* gene found in *Bacillus anthracis* (Figure 1A). Consequently, the gene from *E. faecium* HS0661 was designated *fosB3*.

However, the sequence adjacent to *fosB3* was different from that found in *Staphylococcus* spp. Instead of *rep* genes, which are present downstream from *fosB* on staphylococcal plasmids [17], [18], there was a *tnpA* gene with 86.7% nucleotide identity to a *tnpA* gene located on a plasmid-borne transposable element found previously in *E. faecium* (Figure 1) [19].

Inverse PCR showed that three template DNAs treated with different restriction enzymes produced the same PCR products. No HindIII, BamHI and SmaI sites were found in these products. Using genomic DNA without restriction enzyme treatment as template, the same PCR products were obtained. These results indicate that *fosB3* and *tnpA* were carried by a naturally circular DNA. The circular DNA did not contain a *rep* gene homologue. Its junction region consisted of the left and right inverted repeats and its size was 2435-bp (Figure 1). The circular DNA was also detectable in *E. faecium* strains HS0761, HS07216, and their BM4105RF transconjugants.

### PFGE Type and Plasmid Size

**Figure 2:**
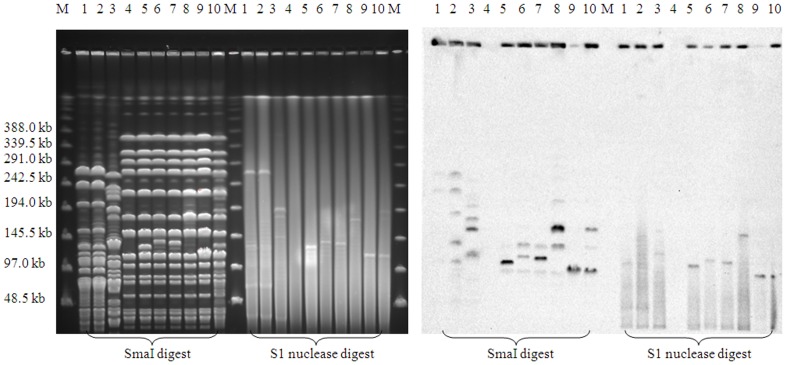
PFGE and Southern hybridization analysis of *Enterococcus faecium* clinical strains, recipient (*E. faecium* BM4105RF), and their respective transconjugants with a *fosB3* probe. Lanes: M, Lambda Ladder PFG Marker (NEB Inc., USA); 1–3, *E. faecium* clinical isolates of HS0661, HS0761 and HS07216, respectively; 4, *E. faecium* BM4105RF; 5 and 6, transconjugants BM0661-1 and BM0661–2; 7 and 8, transconjugants BM0761-1 and BM0761-2; 9 and 10, BM07216-1 and BM07216-2.

The PFGE profiles of SmaI-digested chromosomal DNA divided the 3 clinical *E. faecium* strains into two types. HS0661 and HS0761 were type A, while HS07216 was type B (Table 1). The DNA restriction banding patterns of the transconjugants were similar to BM4105RF. S1 PFGE followed by Southern hybridization analyses with a *fosB3* probe showed that the donor *E. faecium* strains, HS0661, HS0761, and HS07216, carried 3 to 4 different-sized plasmids ranging in size from 35 to 267-kb, and that several plasmid bands were *fosB3* positive. The sizes of *fosB3* positive plasmids in transconjugants ranged from 113 to 135-kb. All were different in size from the plasmids carried by their corresponding donors (Figure 2). With a *vanM* probe, a hybridization signal was only detected at the agarose plug position; no plasmid hybridization with *vanM* was seen.

### Studies in *E. Coli* DH5α

The *fosB3* gene was cloned into pMD19-T and transformed into *E. coli* DH5α. The fosfomycin MIC for the DH5α pMD-fosB transformant was 48 µg/ml while the recipient strain had a fosfomycin MIC of 0.064 µg/ml, indicating that *fosB3* could be expressed in *E. coli*.

## Discussion

We found that resistance to fosfomycin and vancomycin in clinical isolates of *E. faecium* could be co-transferred to *E. faecium* BM4105RF by conjugation, indicating that resistance might be mediated by a mobile element. To identify the fosfomycin resistance determinant, the whole genome of clinical *E. faecium* isolate HS0661 was sequenced by the Solexa method. By comparing with nucleotide sequences deposited in the NCBI database, we found that HS0661 carried a *fosB3* gene, which shared high nucleotide identity with *fosB* genes located on plasmid in *Staphylococcus* spp. 17, 18. Though the sequences flanking *fosB3* from *E. faecium* were different from those downstream from *fosB* on staphylococcal plasmids they had high nucleotide identity with an *E. faecium* plasmid bearing the rRNA methyltransferase gene *emtA* (Figure 1) 19. S1 PFGE and hybridization studies showed that transconjugants carried *fosB3*-positive plasmids (Figure 2). In concert, these findings indicate that *fosB3* is located on a transferable plasmid.

The *fosB3* gene was also detectable by PCR in two other *E. faecium* isolates, HS0761 and HS07216, which were isolated from the same hospital in 2007. Their *fosB3* genes and fosfomycin resistance could be also transferred by conjugation. The PFGE type of HS07216 was different from that of HS0661 and HS0761, also indicating that the *fosB3* gene had transferred to different strains. By hybridization analysis, we found that the sizes of plasmids carried by transconjugants was different from those in their donor strains (Figure 2), suggesting that different recombinational events were involved in the horizontal transfer of *fosB3*.

We found that *fosB3* containing sequence could form circular DNA intermediates. Such circular forms have been previously observed among members of the ISL*3* family, and may be intermediate steps in transposition [20], [21]. Transposition through a circular intermediate has been experimentally confirmed for the ISL*3*-like element, ISPst9 [22]. ISL*3*-like variants are common among enterococcal genomes (both on the chromosome and plasmids), and recombination between plasmids has been frequently observed [23]. The insertion of a *fosB3* gene into Tn*1546* (carrying *vanA*) was recently reported [24]. In this study, circular DNA similar to circular intermediates formed by ISL*3* family elements were observed. Transfer of ISL*3*-like elements may be the cause of the different plasmid sizes found between donors and transconjugants (Figure 2).

When the *fosB3* gene was introduced into *E. coli* DH5α, the fosfomycin MIC increased markedly. This result indicates that *fosB* not only mediate fosfomycin resistance in Gram positive cocci, but could also mediate fosfomycin resistance in Gram negative bacteria.

We are collecting clinical isolates of *E. faecium* from different regions of China to carry out molecular epidemiological studies. Preliminary results show that most high level fosfomycin resistant strains (>1024 µg/ml) are *fosB3* positive. The *fosB3* gene has been detected not only in VanM- and VanA-type vancomycin resistant isolates, but also in some vancomycin-sensitive enterococci (unpublished results).

In conclusion, more attention needs to be paid to the issue of resistance to fosfomycin, especially that mediated by mobile elements, such as plasmids and circular intermediates. Such resistance can spread rapidly, and also contribute to multidrug resistance.
